# Zika virus and pregnancy in Brazil: What happened?

**DOI:** 10.4274/jtgga.2017.0072

**Published:** 2018-03-01

**Authors:** Alessandra Mendelski Pereira, Denise Leite Maia Monteiro, Heron Werner, Pedro Daltro, Tatiana Fazecas, Bianca Guedes, Gabriele Tonni, Alberto Borges Peixoto, Edward Araujo Júnior

**Affiliations:** 1 Department of Obstetrics and Gynecology, State University of Rio de Janeiro, Rio de Janeiro, Brazil; 2 Department of Radiology, Clínica de Diagnóstico por Imagem, Rio de Janeiro, Brazil; 3 Department of Obstetrics and Gynecology, Guastalla Civil Hospital, Reggio Emilia, Italy; 4 Mário Palmério University Hospital, University of Uberaba, Uberaba, Brazil; 5 Department of Obstetrics, Paulista School of Medicine, Federal University of São Paulo, São Paulo, Brazil

**Keywords:** Zika virus, intrauterine infection, microcephaly, ultrasound, magnetic resonance imaging

## Abstract

The recent epidemic of Zika virus (ZIKV) infection in Central and South America is one of the most serious global public health emergencies since the Ebola outbreak in West Africa. In Brazil, especially in the north, northeast, and southeast parts of the country, the ZIKV outbreak is a cause of concern for pregnant women because ZIKV intrauterine infection has been found to be associated with multiple brain malformations and microcephaly. In Brazil, the number of newborns with confirmed microcephaly per year recorded during the ZIKV outbreak, has been approximately 15 times greater than previously reported. Considering that the infection is self-limiting and symptomatic, it is usually diagnosed at the time of routine prenatal scan, especially in the third trimester. In other cases, the disease is detected after childbirth through neuroimaging. This study provides an insight into the history and evolution of ZIKV in Brazil, including current knowledge concerning the transmission, diagnosis, and pathogenesis of the infection. In addition, this review describes the pre- and postnatal neuroimaging findings obtained using ultrasound, magnetic resonance imaging, and computed tomography.

## Introduction

A significant epidemiologic surge of registered cases of newborns affected by microcephaly has occurred in the past two years in Brazil. Further evidence has highlighted a possible association of microcephaly with fever, cutaneous rash, and Guillan-Barré syndrome (GBS) in pregnant women living in areas where Zika virus (ZIKV) is endemic (1,2,3,4). ZIKV was known to cause such symptoms since its discovery in Africa after the second world war raising health attention and concerns following viral spread and outbreak from French Polynesia and Yap island (5) to countries of Central and Latin America, where the virus has shown to be particularly harmful. Until the ZIKV genome was isolated and extracted from the brain cells of a third trimester aborted fetus affected by severe microcephaly in a mother who had a presumed ZIKV intrauterine infection in the first trimester of pregnancy (6), the relationship between viral infection and microcephaly remained a conundrum for healthcare providers. This discovery represents a milestone in the understanding of the infectious disease while confirming a direct pathogenetic and teratogenetic role of ZIKV in cases of congenital infection. However, does ZIKV act as other components of the *Flaviviridae* family or are its teratogenic and neurotropic effects exerted by a different pathogenetic mechanism? Secondly, is there a potential close relationship between ZIKV and specific environmental factors in many areas of Brazil that might have contributed to the degree and severity of congenital ZIKV infection? Some of these crucial questions have been answered by phylogenetic studies, which demonstrated how the Brazilian strain of the ZIKV (ZIKVBR) was genotypically similar in 99% of cases to that isolated in French Polynesia (7). Nonetheless, although a large and rapidly growing number of microcephaly cases were recorded in women with suspected congenital ZIKV infection, serologic demonstration of the virus was only reported in a small subset of mothers (8) due to lack of laboratory tests at the time of the first Brazilian outbreak and availability of such tests in a low-resource clinical setting.

The present review aims to sum up historical and recent insights into the understanding of this severe congenital infection, from epidemiology to diagnosis, prognosis and prevention. Particular results are reported about antenatal and postnatal neuroimaging findings occurring in pregnancy after intrauterine ZIKV infection.

## Etiology

The ZIKV is wrapped by a hicosaedryc capsid where the genome is formed by a single positive RNA helix of 10.794 nucleotides in length. It belongs to the *Flaviviridae* family, *Flavivirus* genus, which includes the yellow fever virus, dengue virus (DENV), and West Nile virus (WNV). 

ZIKV virions are approximately 40-60 nm in size (9) and the viral genome encodes for a single polyprotein of approximately 3400 amino acids, which is further processed into ten different proteins consisting of three structural and seven non-structural proteins (10). It was first identified in a rhesus monkey in 1947 in the Zika forest in Uganda (11). Phylogenetic studies indicate the existence of two strains of the virus: Asian and African. The virus was first detected in humans in Nigeria in 1952 (12). In the following 60 years, benign and sporadic isolated cases of infection in humans were reported in Africa and Southeast Asia. The first outbreak of the disease occurred in the Yap Islands in 2007 where 49 confirmed and 59 suspected cases of ZIKV infections were detected. However, it was estimated that approximately 73% of the population was infected during this outbreak (13). Between 2013 and 2014, more than 28.000 suspected cases of infection were reported in French Polynesia and other Pacific islands. The ZIKV then spread into Brazil, Suriname, and Colombia *via* the Pacific Ocean. Genetic analysis revealed that the virus identified in Brazil belongs to an Asian lineage and originated from the strains from French Polynesia and the nearby islands (14,15). It has been demonstrated that the ZIKVBR in humans may be able to infect the neural progenitor cells (NPCs) causing cell death by inducing apoptosis and autophagy and disrupting the cortical layers leading to microcephaly (7). The history of the ZIKV is summarized in Table 1.

## Transmission

As with other *Flaviviruses*, the transmission cycle of ZIKV between primates and mosquitoes is complex, and man is the occasional unintentional host. The intrinsic period of incubation of ZIKV in human hosts is 4-5 days. During this period, the virus infects other vectors that feed on infected blood, and after an extrinsic incubation period of 8-12 days, the virus is transmitted *via* vector saliva to other hosts (16). The virus is transmitted by mosquitoes of the *Aedes aegypti *species, which are also responsible for transmitting yellow fever, DENV, and chikungunya (CHIKV) fever. ZIKV has also been identified in *Aedes albopictus* mosquitoes (12).

It is noteworthy that *Arboviruses* (mosquito-borne viruses) usually replicate in the cytoplasm of dendritic cells (17), although a different replication mechanism has been hypothesized for ZIKV because its antigens have been observed within cell nuclei (18). It is interesting that the association between intrauterine ZIKV infection and microcephaly/GBS may be the result of the replication of ZIKV in a population with a high *Flavivirus* background- like pre-exposure to DENV infection (19).

The first case to suggest potential sexual transmission of the disease was reported in 2011. In 2015, the second case was reported, and in February 2016, the Centers for Disease Control in the United States recognized sexual transmission as a cause of ZIKV infection. Interestingly, ZIKV RNA can be identified in semen for up to 62 days, although the disease usually develops within 19 days following sexual intercourse (20).

Maternal–fetal transmission is one of the major concerns because ZIKV can cross the placenta at any stage of gestation, causing teratogenic effects (21). It has been confirmed that intrauterine infection occurring during the first trimester of pregnancy is associated with multiple congenital anomalies, mainly affecting the developing brain. The microcephaly risk due to ZIKV infection in the first trimester of pregnancy was estimated as 0.88 to 13.2% in cases of viral infection in the first trimester of pregnancy (22). ZIKV has been detected in the amniotic fluid, placenta, fetal tissues, and abortuses (23). The risk of transmission *via* blood transfusion from infected individuals was confirmed when the ZIKV outbreak occurred in French Polynesia (24).

## Clinical presentations

ZIKV infection is asymptomatic in most cases. The rate of symptomatic individuals among those infected during the Yap Island outbreak in 2007 was 18% (13). There is no difference in the clinical presentation of pregnant and non-pregnant women infected by the virus, and individuals of all age groups are susceptible to the infection. Clinically, the symptoms appear a few days after being bitten by the mosquitoes and are characterized by generalized maculopapular rash, low intermittent fever, conjunctivitis, arthralgia, headache, myalgia, and asthenia. The symptoms have a short duration (2-7 days) and are usually self-limiting; the need for hospitalization is rare (25). In countries where ZIKV has active transmission, an increased incidence of neurologic syndromes, including encephalitis, meningoencephalitis, myelitis, acute flaccid paralysis, and GBS have been recorded (26).

Although ZIKV infection in the mother is accompanied by mild and less severe symptoms, it may cause multiple congenital malformations in the form of prematurity, placental insufficiency, and fetal growth restriction, which may progress to intrauterine fetal demise. Moreover, ZIKV intrauterine infection is associated with an increased number of spontaneous abortions (27).

## Laboratory diagnosis

Laboratory diagnosis is based on the detection of ZIKV RNA using real-time reverse-transcriptase polymerase chain reaction (rRT-PCR) of serum within 5 days from the onset of symptoms. rRT-PCR of serum should be performed after the onset of symptoms because viremia is limited and the decrease in the viral load in maternal blood is very rapid after the onset of rash. The short-term positivity in serum and limited access to tests were the primary contributors to the difficulty in diagnosing the disease during the ZIKV outbreak in Brazil. ZIKV RNA can also be detected in urine with higher titers and for a longer period than in blood (usually within 20 days from the onset of symptoms). ZIKV has also been detected in saliva, semen, and cervical and uterine secretions. However, the suppression period has not yet been determined, and the virus may remain in the urine and semen for several months (11). rRT-PCR can also identify viral RNA in amniotic and cerebrospinal fluids (28).

Serum immunoglobulin (Ig) M antibodies produced by ZIKV infection may be detected from the fifth day from infection using ELISA or immunofluorescence; however, these tests are not specific for ZIKV. Cross-reactivity with other *Flaviviruses* is common and precludes diagnosis in individuals with previous infections including DENV and CHIKV, and those vaccinated against yellow fever (16). Positive IgM antibody tests for ZIKV should be confirmed using the plaque-reduction neutralization test (PRNT), which is specific for ZIKV infection. Currently, this test is not available in Brazil. Although the conventional PRNT test provides a diagnosis of ZIKV infection within 7 days, a recent study reported the development of a rapid PRNT that may provide a diagnosis within 48 hours, without reducing the specificity of the PRNT test (29).

## The disease in Brazil

ZIKV was first diagnosed in Brazil in the State of Bahia in 2015 based on the test results of serum from patients with DENV-like symptoms, including rash, fever, myalgia, arthralgia, and conjunctivitis (30). Later in September, a study reported a significant increase in the number of cases of microcephaly in newborns in northeastern Brazil and later in southeastern Brazil (31). ZIKV RNA was isolated from the amniotic fluid of pregnant women carrying fetuses with confirmed microcephaly and from the brains of fetuses with central nervous system (CNS) malformations (21).

In 2014, 147 cases of microcephaly were reported in Brazil, and in November 2015, the Brazilian Ministry of Health declared a public health emergency of national importance [Emergência em Saúde Pública de Importância Nacional (ESPIN)] because of changes in the pattern of occurrence of microcephaly in Brazil. Between 2015 and 2016, 10.232 cases of changes in the growth and development of newborns possibly due to ZIKV infection and/or overlapping infections were reported. However, the World Health Organization (WHO) did not recognize ESPIN until February 2016. Since March 2016, the number of reported cases of ZIKV intrauterine infection has significantly decreased in Brazil, partly due to the application of a “Rapid Action Strategy” [Estratégia de Ação Rápida (EAR)]. This program strengthens both healthcare and social protection for children with microcephaly. Additional federal funds were allocated to Brazilian States and municipalities to ensure access to diagnostic tests and monitoring the growth and development of these children. The cases of microcephaly registered by EAR were mainly concentrated in the northeast region of Brazil (67.0%), followed by the southeast (20.2%), midwest (5.1%), north (5.0%), and south (2.0%) regions. The States with the highest number of postnatally confirmed cases of microcephaly were Pernambuco (392 cases), Bahia (335 cases), and Rio de Janeiro (234 cases) State (32). In 2016, 215.319 probable cases of fever caused by ZIKV infection were reported in Brazil. Eight neonatal deaths due to ZIKV infection were confirmed through laboratory examination—four in Rio de Janeiro, two in Espírito Santo, one in Maranhão, and one in Paraiba. Although Rio de Janeiro and Piauí State did not report any suspected cases (3), 2347 newborns with microcephaly have been recorded in Brazil as of January 2017.

In 2017, 1653 probable cases of intrauterine ZIKV infection were reported, confirmed postnatally in 275 newborns. The incidence in the northern region of Brazil was higher than that in the other areas. As of February 2017, no cases of neonatal death due to ZIKV infection were confirmed in laboratory examinations (32).

## Fetal infection

The mechanism by which ZIKV crosses the placenta is still unclear, but its neurotropism (6) and ability to destroy neural cells have been clearly studied (33). ZIKV infection induces abnormal mitotic and apoptotic cell death of human NPCs, causing disruptive lesions in the fetal CNS (34). NPCs are the primary target of the ZIKV, and this may partly explain the high number of abnormalities seen in the CNS and detected by neuroimaging examinations (35). The abnormalities affecting the developing brain are often diagnosed during the third trimester of pregnancy (36,37,38) or after birth. The teratogenic effectcs to the embryo, fetus, and newborn caused by ZIKV constitute what has been referred to as congenital ZIKV syndrome. These abnormalities include CNS disorders, impaired development of the eyes and ears, and arthrogryposis (39,40). The most common findings affecting the CNS are microcephaly, ventriculomegaly, brain calcifications, midline echo, and cerebellar defects. When microcephaly is considered, the most severe forms are those following congenital ZIKV infection occurring in the first trimester of pregnancy (41).

Microcephaly is a congenital malformation caused by impaired growth of the fetal brain, which leads to a decrease in the head circumference (HC). The Brazil Ministry of Health recommends an occipito-frontal circumference (OFC) of 32 cm or 2 standard deviation (SD) below the Fenton reference (42,43) for the diagnosis of microcephaly, wheres “severe microcephaly” is defined by an OFC of <3 SD (43,44,45,46). The reference ranges for HC most commonly used in Brazil are those reported in the InterGrowth chart (45,46), although the use of InterGrowth standards reduced the detection of microcephaly compared with Fenton growth chart (43). On November 18th, 2015, the Brazilian Ministry of Health defined newborns with gestational age (GA) of at least 37 weeks with HC smaller than 33 cm at birth as having microcephaly, for which registration is mandatory. However, due to the high numbers of false-positive cases, this cut-off value was reduced to 32 cm on December 9th, 2015 (32).

## The role of ultrasound antenatal management

The International Society of Ultrasound in Obstetrics and Gynecology recommends the following ultrasound (US) monitoring protocols (47):

a) Confirmation of GA preferably before 14 weeks of pregnancy by measuring the crown–rump length;

b) Basic US: basic biometry with evaluation of fetal anatomy and growth rate;

c) Subsequent US: pregnant women with a clinical history of rashes, with or without serologic confirmation, should be scanned every 4-6 weeks;

d) In cases where US shows either fetal HC with 2 SD below the mean expected value for GA and sex or CNS malformations, the mother should be referred to a tertiary care center. A thorough neuroimaging examination should address morphology, integrity, and degree of skull ossification; presence of the cerebral midline echo throughout the length of the skull; and symmetry of the intracranial structures, such as lateral ventricles, cavum septum pellucidum, thalami, cerebellum, and cisterna magna. If available, magnetic resonance imaging (MRI) should be planned to search for other abnormalities affecting the corpus callosum, neural migration, and reduced gyration and sulcation. Other causes of overlapping infections should be discarded. Pregnant woman should be informed of the benefits and risks of amniocentesis to detect ZIKV RNA by rRT-PCR in amniotic fluid.

## Prenatal radiological findings

Detailed knowledge of possible neuroimaging findings in newborns following congenital ZIKV infection is crucial for correct diagnosis, and enhancing parent counseling about the prognosis of the affected child (35).

**Figure 1:**
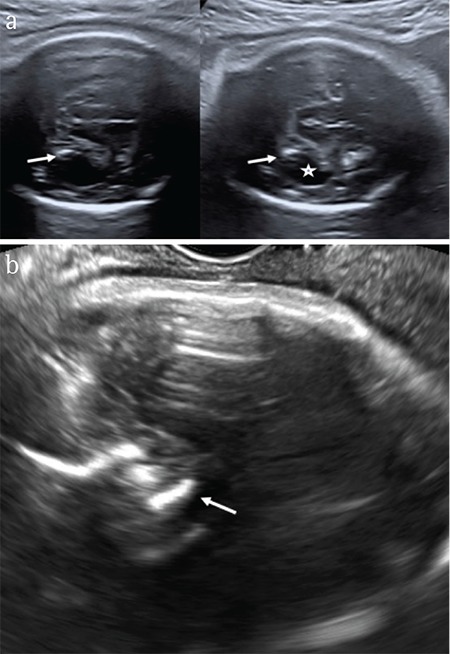
Prenatal ultrasound showing calcifications (arrows), ventricular dilatation (*) and microcephaly. Transabdominal axial plane (34 weeks) (a), ultrasound imaging of the fetal brain during third trimester of pregnancy is hinder by of the ossified skull base. Axial plane obtained by means of transvaginal probe (b) brain calcifications are more visible (arrow)

**Ultrasound: **US is the method of choice for monitoring pregnant women living in areas at increased risk of congenital ZIKV infection. HC is easy to measure, and microcephaly is the most common finding in cases of congenital ZIKV infection. Studies conducted in Brazil to monitor fetal microcephaly using US indicated that microcephaly was severe in 73.7% of patients with HC <5 SD below the mean expected value for GA. Only 10.5% of fetuses had microcephaly alone, whereas 89.5% of fetuses had additional CNS malformations, including periventricular or parenchymal calcifications (63.2%), symmetrical or asymmetrical ventriculomegaly (47.4%), cerebellar abnormalities (42.1%), and cortical atrophy (15.8%). Doppler findings of the umbilical artery of these fetuses were unremarkable (48). Other abnormalities seen were craniofacial disproportion, agenesis or hypoplasia of the corpus callosum, congenital clubfoot, oligohydramnios, cardiac calcification, hepatomegaly, hyperflexion of the second and third fingers, and arthrogryposis. It is important to note that in almost all cases, the US findings were unremarkable until weeks 24, *i.e.*, pathologic findings are usually diagnosed only after GA of 24 weeks. Therefore, a normal morphologic examination *per se* does not rule out the possibility of congenital infection (48). For this reason, pregnant women with proven or suspected congenital ZIKV infection should be followed up with US until delivery (Figure 1).

**Figure 2:**
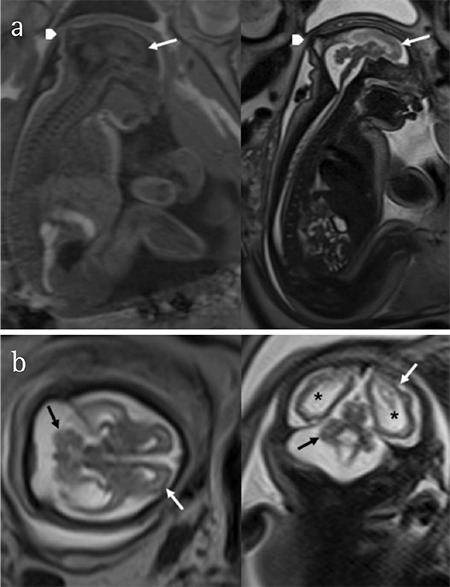
T1- and T2-wieghted magnetic resonance imaging in sagittal plane (37 weeks). Note microcephaly and smoothness of the brain surface (arrow) and redundant skin fold (arrow head) (a), axial and coronal planes showed cortical atrophy (white arrow), ventricular dilatation (*) and cerebellar hypoplasia (black arrow) (b)

**Magnetic resonance imaging:** The MRI findings in fetuses with congenital ZIKV infections are similar to those reported with US. However, brain abnormalities such as polymicrogyria, lissencephaly, pachygyria, abnormal myelination, and changes to white matter and the cerebral cortex, are best evaluated using MRI. However, the sensitivity of MRI in the detection of intraparenchymal brain calcifications is lower than that reported using US (Figure 2) (35).

## Postnatal radiologic findings

**Figure 3:**
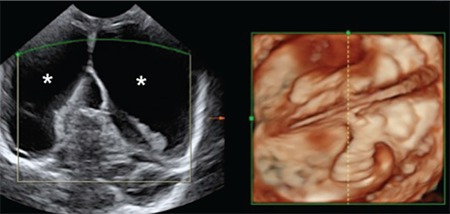
Postnatal transfontanellar ultrasound performed with three-dimensional volume reconstruction showing ventricular dilatation (*)

**Ultrasound:** Transfontanellar US is the examination of choice for the assessment of newborns because of its safety, ease of use, and low cost. It provides adequate visualization of the brain parenchyma and of the ventricular system. US is accurate in the evaluation of the location of cerebral calcifications, and is diagnostic in detecting ventriculomegaly, dysgenesis of the corpus callosum, subependymal cysts, malformations of cortical development, cerebellar and hypoplasia and brain stem (35). Small fontanels or premature closure of cranial sutures as well as bony structures of the skull are common findings in infected children and these factors may hinder US accuracy (Figure 3).

**Figure 4:**
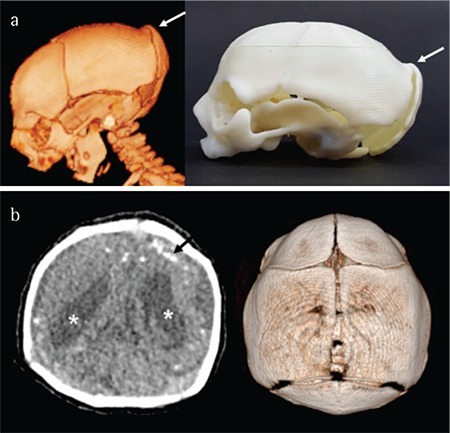
Three-dimensional sagittal reconstruction from computed tomography scan and corresponding three-dimensional printing. The skull has collapsed appearance (arrow) (a), axial plane shows frontal lobe calcifications (arrow), ventricular dilatation (*) with three-dimensional axial reconstruction (b)

**Computed tomography:** Computed tomography (CT) scans provide excellent sensitivity in the assessment of parenchymal calcifications and skull bone deformities, particularly in cases where a three-dimensional reconstruction is used. Intracranial calcifications are clearly identified in CT scans; calcifications may be punctate or coarse and usually located in the corticomedullary junction or immediately below this junction in the frontal and parietal lobe or in the periventricular lobe. Calcifications may rarely occur in the basal ganglia, thalami and cerebellum. Other phenotypic anomalies include craniofacial disproportion, depression of the frontal and parietal bones, overriding of sutures, small fontanels, demyelination, and abnormal density of white matter. Deformities of the cranial bones are secondary to acute cerebral atrophy and reduced intracranial pressure; these findings are commonly seen in infected children (49). The disadvantage of CT, compared with US and MRI, is the increased exposure to radiation (Figure 4).

**Figure 5:**
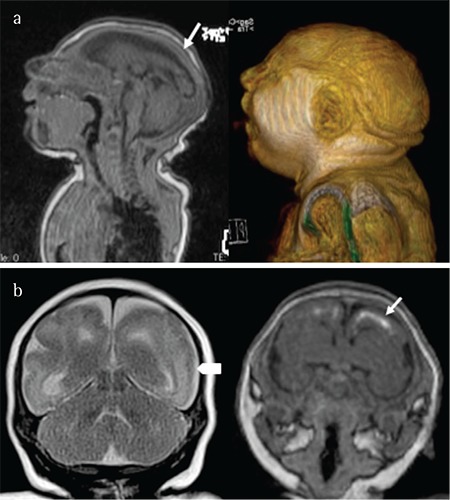
Postnatal T1-weighted magnetic resonance imaging in sagittal plane showing cortical atrophy (arrow) and three-dimensional reconstruction showing microcephaly (a), coronal T2- and T1-weight images showing smoothness of the brain (arrow head) and calcifications (arrow) (b)

**Magnetic resonance imaging:** The quality of MRI in neuroimaging makes it the first choice in the evaluation of children with congenital ZIKV infection. MRI offers high sensitivity and specificity to detecting brain pathology in fetuses and newborns. Defects in the development of the cerebral cortex are associated in almost all cases of microcephaly and are characterized by cortical atrophy and abnormalities in the pattern of the brain gyri (polymicrogyria) and sulci. Demyelination or delayed myelination causes changes in the brain mantle in 88-100% of cases. Moderate-to-severe ventriculomegaly occurs in 85-100% of cases and usually affects the entire ventricular system. Interventricular septa are found in 10-30% of cases, and changes in the corpus callosum (agenesis or atrophy) may affect up to 94% of newborns. Cerebellar hypoplasia may be unilateral or bilateral and affects 27-82% of infected newborns (35). Congenitally-infected children often have sleep disturbances and restlessness requiring sedation before undergoing MRI (Figure 5).

## Congenital infection without microcephaly

Congenital microcephaly may be a marker of intrauterine ZIKV infection but the disease may not always be detected at birth. A Brazilian study involving 13 newborns with congenital ZIKV infection but born without microcephaly documented the presence of other CNS abnormalities, including ventriculomegaly, cerebral atrophy, subcortical calcifications, and cortical malformations, underlining the need to use neuroimaging in these assessments. In these clinical series, the development of microcephaly only presented after birth and was accompanied by severe neurologic disorders, including hypertension, hemiparesis, dystonia, dysphagia, epilepsy, and the persistence of primitive reflexes. These symptoms did not differ from those found in children where microcephaly was detected at birth. However, children born without microcephaly showed better social interaction and made and maintained eye contact and social smiles. However, in this group, 60% of the children had epilepsy and all presented with neuromotor disorders (50).

## Prevention

The most effective prevention strategy is vector control, either by integrated insect management or personal prevention with repellents, long-sleeved clothes, and cooled air. The WHO has also proposed the possibility of using genetically modified mosquitoes to control the populations of *Aedes aegypti*. Since March 2016, academic and pharmaceutical institutions have been working on the development of various types of vaccines against ZIKV, which include the use of live and attenuated viruses (16).

## Treatment

Treatment primarily consists of supportive measures and rest because ZIKV is usually a self-limiting infection, although long maternal viremia has been reported (33). To date, no drugs have been approved for the treatment of ZIKV or other *Flavivirus* infections. A recent *in vitro* study suggested that ZIKV infection could respond to treatment with interferon. However, more studies are needed to confirm this finding (16).

## Prognosis

The biologic effects of ZIKV infection are variable in degree and severity, with different clinical manifestations. The more severe the case of microcephaly, the worse the prognosis is. Early diagnosis allows for early encouragement and improvement of neuromotor performance in children with ZIKV infection.

In conclusion, the outbreak of ZIKV as seen in Brazil in the last two years has contributed to increased numbers of notified cases of microcephaly in newborns; when microcephaly was diagnosed antenatally it was usually at the time of the third trimester scan, and was confirmed, in the vast majority of cases, postnatally by means of CT scans or MRI; antenatal and postnatal diagnosis of microcephaly should be performed according to specific constructed US reference curves; primary microcephaly should be excluded by genetic testing; ZIKV infection as well as overlapping viral infectious diseases (yellow fever, WNV, DENV and CHIKV) and TORCH should be investigated and excluded with diagnostic laboratory testing; amniocentesis to search for viral ZIKV RNA should be recommended after 14-16 weeks in all pregnant women with proven intrauterine ZIKV infection; a thorough US examination, especially a neuroscan, should be performed by expert sonographers in tertiary care centers and fetal MRI incorporated as a complementary neuroimaging investigation; ZIKV has high neurotropism and causes teratogenic effects in the fetus mainly affecting the developing brain; the most severe forms of ZIKV-related congenital infections are associated with prolonged viral shedding and direct placental action is also hypothesized; in targeted cases the ZIKV genome should be sought and isolated from the placenta, and brain tissue and phylogenetic studies should be conducted to investigate the different ZIKV strains; newborns with ZIKV-associated microcephaly should undergo neurodevelopmental outcomes. Although ongoing research is trying to develop specific vaccines against ZIKV, appropriate counselling is advised to prevent congenital infection in pregnant women, especially for those living in epidemic areas of the virus.

## Figures and Tables

**Table 1:**
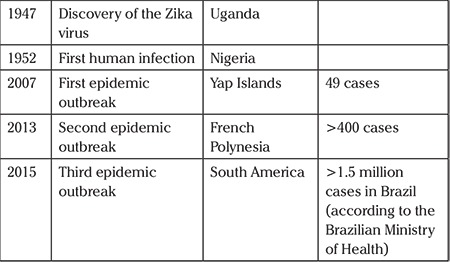
Place of discovery and epidemic outbreak of Zika virus
